# A PHOTOVOICE STUDY OF THE EXPERIENCES OF BIPOC OLDER ADULTS DURING THE COVID-19 PANDEMIC

**DOI:** 10.1093/geroni/igad104.3073

**Published:** 2023-12-21

**Authors:** Angela Ekwonye

**Affiliations:** St. Catherine University, St. Paul, Minnesota, United States

## Abstract

The coronavirus disease 2019 (COVID-19) pandemic intensified the stressful and already difficult circumstances of communities of color. Yet, no current photovoice research has highlighted the lived experiences of low-income Black, Indigenous, and persons of color (BIPOC) older adults during the pandemic. This qualitative study used photovoice to visually portray the struggles of low-income BIPOC older adults and how they recovered from and adapted to the impact of the pandemic. The data analyzed were drawn from interviews, focus groups, photographs, and written stories exploring their experiences and how they found meaning. Results show that participants faced different challenges during the pandemic, such as the fear of COVID-19 exposure and death, struggles to adopt COVID-19 mitigation strategies, loneliness, and social isolation. Amid this crisis of suffering, isolation, and sadness, participants found meaning through a positive reappraisal of the negative situation and engaging in emotional, spiritual, social, and physical self-care practices. The findings have implications for clinical social workers, mental health counselors, faith communities, nurse managers and administrators, and policymakers.

